# Recurrent hyponatremia due to syndrome of inappropriate antidiuresis after traumatic brain injury: two case reports

**DOI:** 10.3389/fendo.2025.1536247

**Published:** 2025-03-06

**Authors:** Iulia Petria, Rita Indirli, Beatrice Mantovani, Valeria Lanzi, Giovanna Mantovani, Emanuele Ferrante

**Affiliations:** ^1^ Endocrinology Unit, Fondazione IRCCS Ca’ Granda Ospedale Maggiore Policlinico, Milan, Italy; ^2^ Department of Clinical Sciences and Community Health, University of Milan, Milan, Italy

**Keywords:** hyponatremia, syndrome of inappropriate antidiuresis (SIAD), traumatic brain injury (TBI), neurohypophysis, tolvaptan, case report

## Abstract

**Background:**

Transient hyponatremia due to syndrome of inappropriate antidiuresis (SIAD) is a frequent (20-50%) complication of traumatic brain injury (TBI), but it rarely persists or recurs. There are only few published reports of patients suffering from non-transient hyponatremia due to chronic SIAD after TBI. We report two more cases with this condition.

**Case 1:**

A 36-year-old woman suffering from major depression and treated with olanzapine reported severe TBI after a severe fall. Following head injury, she developed severe hyponatremia, which was managed with fluid restriction and salt supplementation. Upon hospital discharge, 7 months after trauma, mild hyponatremia was still reported (Na 134 mmol/L), which dropped to severe hyponatremia in a week despite continuation of treatment, and spontaneously returned to normal. Two months later, the patient presented one more episode of moderate hyponatremia without clear triggering events. Pituitary hormones were normal and urinary sodium and urinary and plasma osmolality supported the diagnosis of SIAD. Therefore, tolvaptan 7.5 mg daily was started, with sustained normalization of sodium levels. When olanzapine was stopped, discontinuation of tolvaptan was attempted. However, serum sodium dropped again and tolvaptan had to be resumed, with natremia remaining within normal range at follow-up. Consistently, olanzapine-related hyponatremia could be ruled out and post-traumatic SIAD confirmed.

**Case 2:**

A 37-year-old man experienced TBI with diffuse axonal injury falling during a mountain trip. Over the following year, he presented two episodes of tonic-clonic seizures accompanied by the biochemical finding of moderate-severe hyponatremia. Hyponatremia resolved following hypertonic (3% NaCl) saline infusion, and valproate treatment was started after the second episode. In the following outpatient visits, a progressive decrease of serum sodium from 141 mmol/L to 132 mmol/L was observed, with other tests consistent with SIAD. Therefore, considering the high risk of recurrent seizures as well as the concomitant treatment with valproate, tolvaptan 7.5 mg every other day was started and normal sodium levels have been maintained since then.

**Conclusions:**

We report two cases of recurrent SIAD following TBI, with multiple hyponatremic episodes after initial presentation. This highlights the importance of long-term follow-up of electrolyte abnormalities after head injury.

## Introduction

Neuroendocrine disturbances secondary to traumatic brain injury (TBI) have been reported since the early 1900s (1). Various degrees of anterior hypopituitarism are described (2), as well as posterior pituitary dysfunctions (3). Given that TBI is a growing public health issue (4), potentially associated endocrine dysfunctions deserve particular attention.

In the chronic phase after TBI (at least 3 months after trauma), the prevalence of hypopituitarism ranges between 15 and 50% in different studies, with the most common hormone deficiencies being, in decreasing order of prevalence: growth hormone (GH), adrenocorticotropic hormone (ACTH), gonadotropins (follicle stimulating hormone, FSH, and luteinizing hormone, LH), and thyroid stimulating hormone (TSH) (1). One of the few prospective studies available showed that any degree of hypopituitarism was present in 33% of victims of TBI at 3 months, with this percentage decreasing to 12% at 12 months (5).

In the early phase following TBI, neurohypophysis dysfunctions are common, with consequent imbalance in plasma sodium levels: in particular, hyponatremia affects 20–50% of patients and is mainly caused by syndrome of inappropriate antidiuresis (SIAD) (3). Hypernatremia due to vasopressin deficiency (central diabetes insipidus) is likewise described in the acute phase of TBI, with a reported incidence of 3–26% (6).

In general, hyponatremia is the most common disorder of body fluid and electrolyte balance. It varies in severity, as assessed biochemically by serum sodium concentration (“mild”, between 130 and 135 mmol/L, “moderate”, between 125 and 129 mmol/L, and “severe”, below 125 mmol/L), and clinically by the presence of symptoms [“moderately severe”, accompanied by nausea without vomiting, confusion, or headache, and “severe”, with vomiting, cardiorespiratory distress, somnolence, seizures, or a Glasgow Come Scale (GCS) score ≤ 8] (7). Even when asymptomatic, natremia below 135 mmol/L is associated with increased morbidity and mortality (8). While acute hyponatremia (defined as hyponatremia that is documented to exist <48 hours) is mainly responsible for neurological manifestations, chronic hyponatremia is associated with a greater risk of falls, osteoporosis, fractures, gait instability, and cognitive decline, as well as prolonged hospitalization (9). As regards TBI, the neurological manifestations of hyponatremia and acute brain injury are quite similar, therefore differentiation may be difficult. Moreover, correlation between hyponatremia occurrence and trauma severity is conflicting. Trauma severity can be assessed both clinically, e.g., with presenting GCS score, and by imaging, e.g., according to computed tomography (CT) abnormalities. On the one hand, it appears that mild and moderate head injuries are complicated by hyponatremia more frequently than severe head traumas; on the other hand, CT grading of neurological damage seems to better correlate with severity and occurrence of hyponatremia (10).

Hyponatremia due to SIAD following TBI is generally mild and transient. Born et al. (11) suggested a distinction between early syndrome and late syndrome: the former is rarer, with an onset of SIAD between the second and fourth day following TBI, less severe neurological dysfunction, and mostly associated with skull base fractures; the latter is more common, with an onset of SIAD between the seventh and the nineteenth day after TBI with a peak of incidence on the ninth day, more extensive neurologic dysfunctions, and moderate-severe degrees of hyponatremia. While the early syndrome is primarily related to encephalic trauma lesions, the late syndrome is presumably multifactorial, also connected to intensive care procedures (11). Lohani and Devkota (10) describe a clustering of cases at the end of the first week and at the beginning of the second week, with a mean duration of 1.78 (1-3) days.

There are only few published reports of patients suffering from persistent or recurrent hyponatremia due to chronic SIAD after TBI. We report two additional cases with this condition, highlighting the need for follow-up in these patients.

## Case presentation

### Case 1

A 36-year-old woman was referred to our Centre for chronic hyponatremia. She was affected with hypothyroidism secondary to total thyroidectomy for toxic multinodular goiter, allergic asthma and major depression. Eight months earlier, she had a major trauma following a fall from a first-floor window. She reported severe TBI with multiple cerebellar, frontobasal, and temporal lacerated-contused foci, diffused subarachnoid hemorrhage, multiple fractures of skull and facial massif, fracture of L1 vertebra and ribs and pulmonary and liver contusion. The patient underwent multiple neurosurgical interventions (osteodural decompression, cranioplasty, and vertebral stabilization). During her seven-month hospital stay, she developed hyponatremia, which was managed with fluid restriction and oral salt supplementation. Therapy at discharge comprised oral salt supplementation (NaCl 1 g three times daily), levothyroxine 100 µg daily, pregabalin 25 mg twice daily, olanzapine 1.25 mg twice daily, and paracetamol 500 mg daily, which guaranteed adequate pain control. Plasmatic sodium level on discharge was still compatible with mild hyponatremia (Na 134 mmol/L), dropped to severe hyponatremia in a week (Na 123–121–123 mmol/L) despite continuation of treatment, and spontaneously returned to normal (Na 133–134–135 mmol/L). Plasma potassium level remained within limits. Follow-up computed tomography (CT) scan showed no intracranial hemorrhages, a very thin left fronto-temporal subdural hygroma, and a frontal malacic gliotic focus.

When the patient came to our attention one month after discharge, she presented normal serum sodium (Na 142 mmol/L). On physical examination, blood pressure was 110/60 mmHg with a heart rate of 60 beats per minute. Reported fluid intake was of 1000 mL daily, while urinary output was about 1250 mL per day. The assessment of pituitary hormones documented normal basal and stimulated anterior pituitary function (**Table 1**). Kidney function, full blood count, and blood glucose were also within normal ranges. Normal natremia was confirmed (Na 140 mmol/L), while spot urine analysis revealed urinary sodium excretion (U-Na) of 192.1 mmol/L, urinary potassium excretion (U-K) of 106.2 mmol/L, and urinary specific gravity of 1025. Therefore, transient SIAD secondary to TBI was hypothesized. Given the stability of normal sodium plasma levels, withdrawal of salt supplementation was attempted.

**Table 1:** Patients’ exams at initial diagnosis of SIAD (syndrome of inappropriate antidiuresis) in the two cases presented.

Analyte	Value – Case 1	Value – Case 2	Normal range
Plasma sodium	140 mmol/L	131 mmol/L	135-145
Plasma potassium	4.5 mmol/L	4.7 mmol/L	3.5-5.5
Urinary sodium	192.1 mmol/L	174 mmol/L	
Urinary potassium	106.2 mmol/L	47 mmol/L	
Urinary osmolality	n.a. ^1^	958 mOsm/kg	50-1200
Urinary specific gravity	1025	n.a.	1005-1030
Glycemia	84 mg/dL	n.a.	70-100
Creatinine	0.75 mg/dL	0.72 mg/dL	0.72-1.18
Urea	n.a.	26 mg/dL	15-55
Plasma uric acid	n.a.	3.0 mg/dL	3.5-7.2
Hemoglobin	13.4 g/dL	13.5 g/dL	11.6-15.0 (case 1) 13.2-16.6 (case 2)
Adrenocorticotropic hormone (ACTH)	12 ng/L	12 ng/L	< 46
Insulin-like growth factor 1	217 µg/L	170 µg/L	60-200 (case 1) 90-239 (case 2)
Thyroid stimulating hormone	1.700 mIU/L	1.070 mIU/L	0.280-4.300
Free Thyroxine	20.3 ng/L	16.9 ng/L	8-17 (case 1) 12-22 (case 2)
Prolactin	11.5 µg/L	11.3 µg/L	1.7-16.0
Basal cortisol	5.77 µg/L	12.8 µg/L	4.8-15.8
Peak cortisol after 1 µg intravenous ACTH stimulation test	20.3 µg/L	n.a.	> 13.6 (26)

^1^n.a., not available.

However, one month after treatment discontinuation, the patient developed moderate hyponatremia again (Na 127 mmol/L), with low plasma chloride (95.8 mmol/L, n.v. 96-108), normal plasma potassium (K 4.5 mmol/L), and optimal levothyroxine replacement therapy (TSH 2 mIU/L). She did not report any symptom of hyponatremia, and remained clinically euvolemic. Low plasma sodium levels were subsequently confirmed (Na 129–126.6 mmol/L), and urinary sodium and urinary (U-Osm) and plasma osmolality (P-Osm) supported the diagnosis of moderate euvolemic hypotonic hyponatremia (P-Osm 262 mOsm/Kg, U-Osm 625 mOsm/Kg, U-Na 93.2 mmol/L, U-K 38.5 mmol/L). Treatment with tolvaptan 15 mg daily was started. One week afterwards, natremia was normalized (Na 138 mmol/L). The patient remained euvolemic and reported an increase in thirst and urinary output (2500 mL daily). Tolvaptan dosage was reduced to 7.5 mg daily and then to 7.5 mg every other day, and sodium levels remained normal at follow-up.

**Figure 1:**
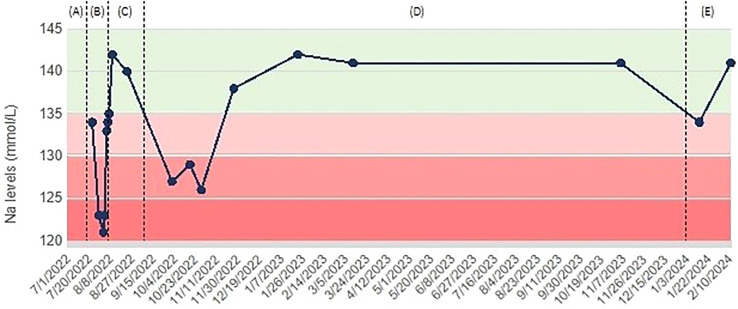
Case 1: trend of serum sodium levels over time, with associated treatments. **(A)** Severe hyponatremia following TBI (natremia value unavailable), managed with fluid restriction and oral salt supplementation; **(B)** mild hyponatremia at hospital discharge, dropping to severe hyponatremia, despite continuation of fluid restriction and oral salt supplementation; **(C)** Spontaneous return to normal natremia, so treatment withdrawal was attempted; **(D)** Recurrence of moderate hyponatremia; tolvaptan was started; **(E)** mild hyponatremia after tolvaptan withdrawal; eventual reintroduction of tolvaptan and subsequent maintenance of normal natremia.

Ten months afterwards, on neurological indication, treatment with pregabalin and olanzapine was withdrawn, and after 45 days natremia still remained within range (Na 141 mmol/L). Therefore, an attempt was made to stop tolvaptan, however one month later mild hyponatremia recurred, with plasma sodium dropping to 134 mmol/L. This excluded drug-induced hyponatremia and confirmed post-TBI SIAD as the most likely diagnosis. Tolvaptan 7.5 mg every other day was reintroduced, and natremia at one-month follow-up was 141 mmol/L (**Figure 1**).

### Case 2

A 35-year-old man came to our attention for a previous episode of severe hyponatremia, documented when he presented to the emergency department with tonic-clonic seizures (Na 124 mmol/L, Cl 86 mmol/L, U-Na 28 mmol/L). Over the preceding 16 hours he reported nausea, however he maintained regular food and fluid intake of about 2.5 L daily as usual, and denied any vomiting. He was not on any medications when he was admitted to hospital. His medical history comprised a severe concussive cranioencephalic trauma after a fall during a mountain hike the previous year.

Following seizures, he was hospitalized to complete the diagnostic work-up. Brain magnetic resonance revealed post-traumatic diffused axonal injury, with cortical and subcortical hemosiderin residues. Pituitary imaging was normal, without evidence of signal alteration, except for a minimal thinning of the terminal portion of the stalk, with inhomogeneous enhancement; the neurohypophysis was recognizable. Hyponatremia promptly responded to infusion of hypertonic (3% NaCl) saline solution, and the patient was prescribed fluid restriction of 1200 mL daily. Exams during hospital stay were consistent with SIAD (**Table 1**). He was discharged with the prescription of fluid restriction, neurological follow-up, and no specific medications.

On our first visit, one month later, he displayed normal natremia (Na 143 mmol/L). On physical examination, he appeared euvolemic, and blood pressure was 110/70 mmHg. The previous symptomatic episode of hyponatremia was then deemed transient and two alternative differential diagnoses were considered: either SIAD following TBI, or hyponatremia secondary to the release of antidiuretic hormone during nausea and subsequent acute dilutional hyponatremia. Given the normalization of plasma sodium levels, an attempt was made to discontinue fluid restriction, and the patient suffered no further overt hyponatremic episodes over the subsequent months. Indeed, the following sodium levels were recorded: 135 mmol/L (4 months after the first episode), 137 mmol/L (at 5 months), 135 mmol/L (at 6 months), 142 mmol/L (at 10 months).

**Figure 2:**
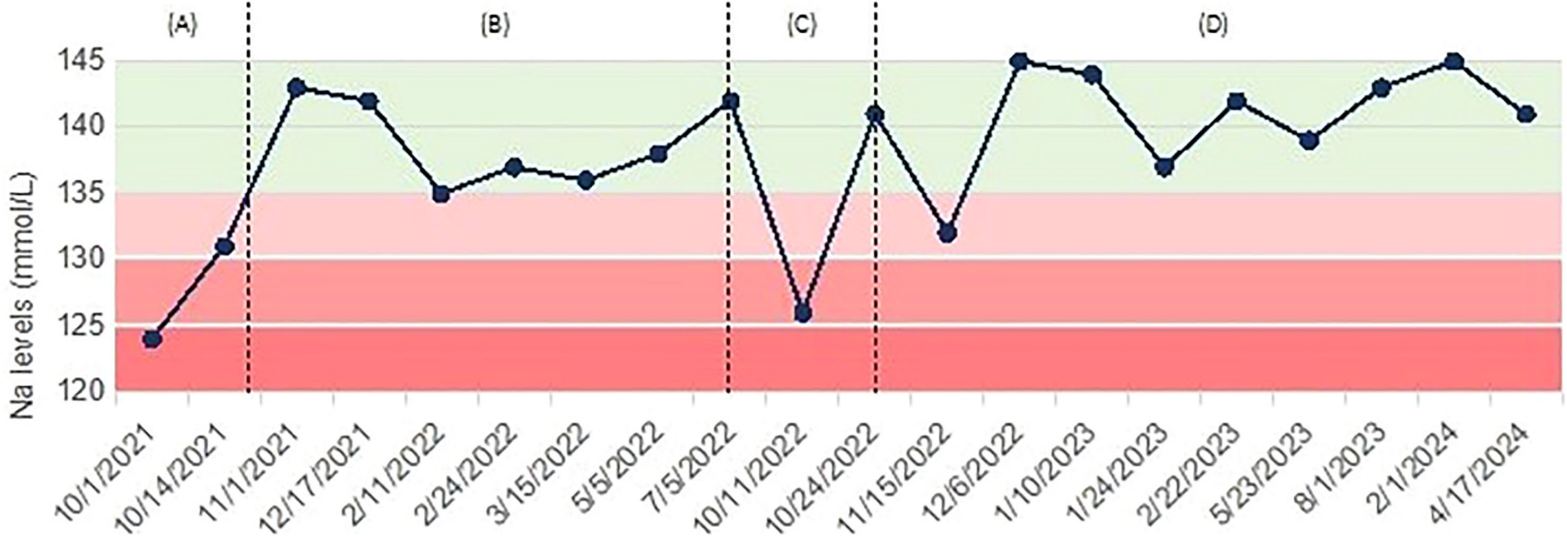
Case 2: trend of serum sodium levels over time, with associated treatments. **(A)** Severe hyponatremia presenting with tonic-clonic seizure, managed with hypertonic (3% NaCl) saline; **(B)** maintenance of normal natremia, no treatment; **(C)** recurrence of moderate hyponatremia, treated with hypertonic (3% NaCl) saline; **(D)** persistence of mild hyponatremia and start of tolvaptan.

However, the following year he presented a second episode of seizures, not preceded by other symptoms, including nausea, vomiting, or diarrhea. No clear triggers for hyponatremia development could be identified. Electrocardiogram was normal. Moderate hyponatremia (Na 126 mmol/L) was observed on this occasion, therefore hypertonic (3% NaCl) saline infusion was administered, with gradual increase of serum sodium (from 128 to 133.1 mmol/L). Chronic treatment with valproic acid 300 mg twice daily was started on neurological indication. This new episode supported the diagnosis of post-TBI SIAD, with seizures being likely facilitated by hyponatremia in a patient with a reduced epileptogenic threshold. Given that valproate is a known possible pharmacological cause of hyponatremia (8), the patient was prescribed frequent control of plasma sodium levels. In the following visits, a progressive reduction of natremia from 141 mmol/L to 132 mmol/L was observed. In particular, the patient reported asthenia, agitation, and dizziness during the days preceding the occurrence of moderate hyponatremia, but he denied any nausea, vomiting, diarrhea, or excessive fluid intake. Therefore, considering the high risk of recurrent seizures, as well as the concomitant treatment with valproate, tolvaptan 15 mg daily was initiated. While on therapy, plasma sodium rose to 145 mmol/L and patient reported excessive thirst and increased fluid intake (from 2000 mL to 3000 mL daily). For these reasons, tolvaptan was gradually down-titrated to 7.5 mg every other day, and normal sodium levels have been maintained. Antiepileptic treatment with valproate was replaced with lacosamide 100 mg twice daily, while natremia remained normal and the patient reported no further seizures (**Figure 2**).

## Discussion

We describe two cases of relapsing hyponatremia secondary to SIAD following TBI. Potentially confounding factors have been addressed in both cases (pharmacologic-induced hyponatremia due to olanzapine in the first one, and epilepsy treatment in the second one).

SIAD is a heterogeneous condition. In general, the most frequent etiologies include malignancies (e.g., small-cell lung carcinoma), disorders of the central nervous system (e.g., vascular insults), medications (e.g., proton-pump inhibitors, antidepressants) (12), pulmonary disorders (e.g., pneumonia), transient conditions (e.g., nausea, pain), and genetic causes (e.g., activating mutations in the vasopressin 2 receptor) (9). Mechanisms of onset of SIAD after TBI are not fully elucidated. It has been hypothesized that damage to the pituitary stalk or to the posterior pituitary, as well as a disorganized activity of the hypothalamic osmoreceptors and of the brainstem cardiovascular centers, may lead to inappropriate non-osmotic release of antidiuretic hormone (13, 14). TBI-associated pain and stress also concur to stimulate antidiuretic hormone secretion (7). While this is generally a temporary effect (10), as in most cases reported hyponatremia resolves after a brief period of fluid restriction, our cases support the possibility that the damage may persist, and hyponatremia may relapse. The second case described herein highlights that the inappropriate release of antidiuretic hormone with subsequent hyponatremia development can be erratic and recur even after a prolonged interval, complicating the clinical management of these patients.

Actually, hyponatremia after TBI rarely persists or recurs, in the absence of secondary causes: Agha et al. (15) describe a series of 102 TBI patients, with chronic SIAD occurring only in two cases (one with concomitant obstructive hydrocephalus, and the other with treatment with selective serotonin reuptake inhibitor citalopram).

The other reports available of non-transient, i.e., withstanding or recurrent hyponatremia following TBI are summarized in **Table 2** (16–20). Of these, two reported a relapsing trend (19) (20), and only one was managed with chronic tolvaptan treatment (20). Overall, there is little data available in literature about long-term follow-up of electrolyte balance in patients suffering from TBI; our case reports contribute to this field. As a general indication, we suggest monitoring electrolytes monthly during the first six months after TBI, and subsequently every 3-6 months according to clinical progress.

**Table 2:** Summary of case reports of non-transient (i.e. persistent or relapsing) hyponatremia after traumatic brain injury (TBI), with treatemnt and outcomes.

First Author	Journal^1^, Year^2^	Type	Primary and Concomitant Conditions	Hyponatremia Severity^3^	Onset of Hyponatremia after TBI	Treatment	Outcome(s)
Van der Voort, S, et al.	The Netherlands Journal of Medicine, 2020	Case Report	TBI after falling down the stairs, with consequent bilateral mastoid fracture, paralysis of the facial nerve, central skull base fracture	Severe (114 mmol/L)	Two weeks	At onset: fluid restriction and hypertonic (3% NaCl) saline; subsequently: fluid restriction and oral sodium chloride plus urea powder	Spontaneous resolution of withstanding SIAD five and a half years after TBI
Dick, M, et al.	Endocrinology, Diabetes and Metabolism, 2015	Case Report	TBI after a high-speed motorbike accident, with associated complications of post-traumatic amnesia and mild cognitive deficits	Severe (117 mmol/L)	Three days	At onset: fluid restriction; subsequently: due to difficult adherence to fluid restriction, start of Demeclocycline 300 mg twice daily	Persisting SIAD, with normalization of sodium levels only after start of demeclocycline (four years after TBI)
Graziani, C, et al.	Journal of Neurology, Neurosurgery and Psychiatry, 2012	Short Report	Severe TBI with multiple lacerative cerebral lesions and significant perilesional brain edema after an assault; two weeks later, meningoencephalitis	Severe (119 mmol/L)	Two weeks	At onset: antibiotics (ceftazidime and vancomycin) and hypertonic (3% NaCl) saline; subsequently: tolvaptan 15 mg daily	Chronic tolvaptan-dependent SIAD: normalization of natremia with start of tolvaptan, but relapse of hyponatremia on discontinuation attempt
Chang, CH, et al.	American Journal of Medical Sciences, 2008	Case Report	TBI with minimal bilateral frontal subdural hematoma after an accidental fall; two months later, relapse of hyponatremia concomitantly with surgery for a lumbar spine compression fracture; four months after TBI, second relapse after surgery for a displaced transpedicle screw	At onset: severe (122 mmol/L); at first relapse: severe (121 mmol/L); at second relapse: moderate (128 mmol/L)	Onset: four days; first relapse: two months; second relapse: four months	At onset: fluid restriction and hypertonic (3% NaCl) saline; at relapses: fluid restriction only	Recurrent SIAD-related hyponatremia, with prolonged recovery of the initial episode; no further occurrence of hyponatremia
Kumar, PD, et. al.	Annals of Internal Medicine, 2001	Letter to Editor	TBI with bilateral frontal lobe contusions and subarachnoid hemorrhage after an assault; one year later, generalized tonic-clonic seizures with encephalomalacia on the frontal lobes on CT scan	Severe (119 mmol/L)	One year	Fluid restriction	Late-onset SIAD resolving with fluid restriction; no further occurrence of seizures

^1^Published in English in international journals. ^2^Published after the year 2000. ^3^Mild (130 to 135 mmol/L), moderate (125 to 129 mmol/L), severe (≤ 124 mmol/L).

Antipsychotic use may be associated with reporting of hyponatremia; however, it is difficult to estimate the real incidence of this adverse drug reaction based on existing literature data (21). Periodic monitoring of natremia should be maintained, especially after start of therapy, and when additional risk factors for hyponatremia co-exist. Moreover, some antipsychotics, including olanzapine, have also been reported to have a beneficial effect on polydipsia and hyponatremia in schizophrenic patients (22). In the first case we report on, natremia remained normal after olanzapine discontinuation but dropped with subsequent tolvaptan withdrawal attempt, thus supporting post-traumatic SIAD as the most likely diagnosis.

Antiepileptic drugs are also known to be associated with hyponatremia: in particular, carbamazepine use was the most important risk factor for the development of hyponatremia in a large cohort of adult epileptic patients, and add-on valproate treatment increased the risk (23). A recent review of case reports concluded that valproate-associated SIAD is a rare phenomenon, still caution is warranted for the occurrence of hyponatremia (24).

Management of hyponatremia depends on the underlying cause, severity (as indicated by the presence of neurological symptoms), and speed of onset (7). Acute symptomatic hyponatremia is generally corrected with hypertonic saline (NaCl 3%). Chronic hyponatremia management needs a controlled and limited correction, in order to avoid the neurological complications of osmotic demyelination (17). According to European clinical practice guidelines for hyponatremia (7), fluid restriction is recommended as first-line treatment in chronic hyponatremia without severe or moderately severe symptoms, while the following can be considered equal second-line treatments for SIAD: increasing solute intake with 0.25–0.50 g/kg per day of urea or a combination of low-dose loop diuretics and oral salt supplementation. Both cases we describe were eventually treated with tolvaptan, an inhibitor of the receptor 2 of the antidiuretic hormone, which decreases the expression of aquaporin on renal collecting duct cells, thereby promoting free water clearance (20). Response in terms of rise in natremia is generally rapid, therefore caution to use the lowest effective dose of the drug should be used, in order to avoid overcorrection and reduce the risk of osmotic demyelination (25). For patients necessitating long-term treatment with tolvaptan, periodic attempts of discontinuation, e.g., every 1 or 2 years, can be considered, with tight monitoring of sodium levels.

## Conclusion

The two cases reported here emphasize that hyponatremia secondary to SIAD may be a persistent or recurrent complication after TBI. Symptoms of neuroendocrine dysfunctions following TBI might be subtle, therefore clinical suspicion needs to remain high, and monitoring of plasma sodium levels should be maintained even longer than the acute phase. Maintaining an appropriate treatment for SIAD could be considered, in order to prevent recurrence of hyponatremia and avoid further risk.

## Patient perspective

### Case 1

“My experience with hyponatremia began after a serious accident I suffered almost three years ago, when I fell from the window of my first-floor home while I was closing a shutter. I reported a severe head injury, a burst vertebral fracture, and several rib injuries. I was hospitalized for about a month, undergoing a decompressive craniotomy and a reduction of the vertebral fracture. Subsequently, I followed a rehabilitation program of approximately seven months.

The referral to the Policlinico Hospital in Milan was suggested by a doctor at the rehabilitation clinic, for an evaluation of pituitary function in the context of chronic hyponatremia, which worsened and had been managed with intravenous saline solutions and salt tablets up to that point.

Following the initial visit, the salt tablets were discontinued to monitor sodium levels. Subsequently, given the declining condition, I was prescribed the drug tolvaptan, a treatment that has continued effectively to date and has allowed me to keep an important issue under control. I am currently in follow-up for the continuation of therapy and periodic check-ups.”.

### Case 2

“Sodium, for common people, is only famous because of water brand advertisements.

My approach with hyponatremia happened 3 years ago, following my first epileptic seizure, probably due to a thickening in the pituitary stalk after a severe traumatic brain injury due to a fall while hiking in the mountains.

Before that moment, I always considered myself “a heavy drinker” of water, however afterwards I had to get used to the fact that I would only be so on the days I take my medicine (tolvaptan), with subsequent very frequent urination (up to twenty times within four hours initially), even at night.

After two years, I think I have reached a stable situation, I drink and urinate regularly; I take in less fluids than before, still I live my days without rushing to the toilet, as used to happen before.

I am not sure whether it is subjective, but lately, on the days I take tolvaptan, I feel like still water does not quench my thirst as before; I prefer drinking sparkling water and even fizzy drinks.”

## Data Availability

The raw data supporting the conclusions of this article will be made available by the authors, without undue reservation.
